# High Expression of TBC1 Domain Family Member 22A is Related to Poor Prognosis in Ovarian Serous Cystadenocarcinoma

**DOI:** 10.7150/ijms.99744

**Published:** 2024-10-07

**Authors:** Xiaofeng Lv, Ruyue Gong, Lili Guo, Changyu Wang

**Affiliations:** 1 Department of Obstetrics and Gynecology, Tongji Hospital, Tongji Medical College, Huazhong University of Science and Technology, Wuhan, 430030, China.; 2 Cancer Biology Research Center, Tongji Hospital, Tongji Medical College, Huazhong University of Science and Technology, Wuhan, 430030, China.; 3 Department of Obstetrics and Gynecology, The Third Affiliated Hospital of Zhengzhou University, Zhengzhou, 450052, China.

**Keywords:** TBC1D22A, prognosis, TCGA, ovarian serous cystadenocarcinoma, immune cell infiltration.

## Abstract

**Objective:** TBC1 domain family member 22A (TBC1D22A) possesses GTPase-activating protein (GAP) activity of Rab family proteins and has not been reported in ovarian serous cystadenocarcinoma (OSC). The research was designed to evaluate the expression and prognostic effect of TBC1D22A in OSC.

**Methods:** TCGA, GTEx, GEO, HPA, and GDSC databases were adopted to explore the oncogenic mechanism of TBC1D22A in OSC, as well as the correlation between TBC1D22A and patient prognosis, IC50, stemness index, immune checkpoint, and immune infiltration. To compare the occurrence of end-point times, Kaplan-Meier survival curves were used. Independent prognostic factors of patients with OSC were analyzed with both univariate as well as multivariate Cox regression analyses, and the overall survival (OS) of the patients at 1, 2 and 3 years was predicted with nomograms.

**Results:** TB1D22A expression was elevated in OSC, and high expression of TBC1D22A was related to poor OS, progression free survival (PFS), disease specific survival (DSS), and disease-free survival (DFS) in OSC. TBC1D22A had predictive value in both univariate and multivariate Cox regression analysis. TBC1D22A was positively correlated with M2 macrophage infiltration and the expression of most immune checkpoint genes. IC50 for cisplatin and paclitaxel increased in patients with overexpression of TBC1D22A.

**Conclusion:** TBC1D22A is an independent prognostic risk factor for patients of ovarian cancer. Future research is required to fully understand the carcinogenic mechanism and clinical utility of TBC1D22A in ovarian cancer.

## Introduction

GAPs generally contain a conserved protein domain, Tre2-Bub2-Cdc16 (TBC). This domain was first discovered in yeast cells and is the common conserved domain of the oncogene Tre-2, the yeast cell cycles regulatory proteins Bub2 and Cdc16^1^. TBC1D22A is localized to the Golgi apparatus and has two important catalytic residues, the arginine finger and the glutamine finger, which drive the hydrolysis of Rab-GTP to Rab-GDP and release it into the cytoplasm^2^. According to previous studies, TBC1D22A is associated with recessive genetic epilepsy^3^, schizophrenia^4^, and liver cancer^5^. Interestingly, the role of TBC1D22A in gynecological tumors has not been reported.

Ovarian cancer shows a 5-year survival rate of 48.6% as the most lethal gynecological malignancy^6^,^7^. OSC acts as the most common and aggressive subtype of ovarian cancer, taking up 67.5% of ovarian cancer subtypes^8^. Most individuals with ovarian cancer are identified at an advanced stage and have a poor prognosis because the disease lacks noticeable signs and is easily metastasized in its early stages^9^. NCCN guidelines recommend primary debulking surgery in addition to platinum-based chemotherapy as the first choice for patients with OSC 10. However, most patients with OSC still experience tumor recurrence after treatment. The recurrence rate of patients within 6 months can reach 25%, the median PFS is only 18 months, and recurrence is considered the main cause of death in these patients^11^. Therefore, we need to explore more biomarkers for the evaluation of the prognosis and the adjustment of postoperative treatment in patients with ovarian cancer.

Previously, we performed whole-exome sequencing on tissue samples from a cohort of patients with ovarian cancer and found that platinum-sensitive patients show a significantly higher missense mutation rate of TBC1D22A than platinum-resistant patients. This study thoroughly investigated the association between TBC1D22A expression in OSC and prognosis, immune infiltration, stemness index, immune checkpoints, and chemotherapy drugs. The research was aimed at evaluating whether TBC1D22A is an independent prognostic factor in ovarian cancer.

## Methods

### Cell culture, chemicals, and antibodies

The China Center for Type Culture Collection (Wuhan, China) offered the human ovarian cancer cell lines COC1, ES-2 and SKOV3, and the human normal ovarian epithelial cell line IOSE-80 was provided by the Cancer Biology Research Center at Huazhong University of Science and Technology (Wuhan, China). SKOV3 and ES2 cells were cultured using McCoy's 5a. 1640 medium was used for IOSE80 and COC1 cells. The primers below were adopted for TBC1D22A: 5'-TTTGATCCACTGTTACATGGCAC-3'(forward) and 5'-TCCCAGGCATCGCTGTGT-3'(reverse). β-actin: 5'-CATGTACGTTGCTATCCAGGC-3'(forward) and 5'-CTCCTTAATGTCACGCACGAT-3' (reverse). Western blot analysis employed the antibodies below: TBC1D22A (18332-1-AP, Proteintech, Wuhan, China), GAPDH (60004-1-Ig, Proteintech, Wuhan, China).

### Differential expression analysis

The extraction of RNA-sequencing expression profiles, clinical data and survival information of OSC patients from The Cancer Genome Atlas (TCGA) database was made^12^. The genotype-tissue expression (GTEx) database provided RNA-sequencing data of normal ovarian tissue 13. Furthermore, differential expression of TBC1D22A was validated in two ovarian cancer datasets (GSE40595 and GSE66957) from the GEO database^14^. Difference in TBC1D22A mRNA expression in ovarian cancer was analyzed by Wilcox-tests. The R package "ggplot2" was adopted to design the box plot. Experimental procedures for reverse transcription-quantitative polymerase chain reaction (RT-qPCR) and Western blot analysis were carried out as mentioned previously^15^.

### Immunohistochemistry staining

The HPA database offered the immuno-histochemical findings of TBC1D22A in ovarian cancer and normal ovarian tissues to assess the discrepancies in TBC1D22A expression at the protein level. The antibody number was HPA078026. Twenty-two pairs of ovarian cancer tissues and adjacent tissues were obtained from the Department of Obstetrics and Gynecology, Tongji Hospital, Tongji Medical College, Huazhong University of Science & Technology. According to the platinum-free interval, 13 patients were diagnosed as platinum-resistant and 9 patients were diagnosed as platinum-sensitive. Experimental procedures were performed as described previously^15^, and the antibody used was TBC1D22A rabbit polyclonal antibody (18332-1-AP, Proteintech, Wuhan, China). The present study was supported by the Ethics Committee of Tongji Hospital in accordance with the ethical standards in the Declaration of Helsinki (TJ-IRB202402029).

### Recognition of prognostic factors in OSC

We evaluated the prognostic value of TBC1D22A, race, age, pathological grade, and TNM stage in ovarian cancer using univariate and multivariate Cox regression analyzes. Forest plots were adopted through the "forestplot" package to show *p*-*values*, hazard ratio (HR), and 95% confidence interval (CI) for every variable. According to the outcomes of multivariate Cox regression analysis, the 1,2, and 3-year survival rates was predicted by establishing a nomogram with the "rms" package. Providing a graphical result of these factors, the nomogram can be adopted to calculate the prognostic risk for a patient by the points related to each risk factor.

### Correlation between TBC1D22A and the survival of OSC

We extracted survival data for every sample from the TCGA database. To evaluate the correlation of TBC1D22A expression with the prognosis of patients with OSC, several key indicators were selected: OS, PFS, DFS, and DSS. Based on patient survival information, we plotted Kaplan-Meier curves using the R packages "survminer" and "survival". The association of TBC1D22A with survival was decided by calculating the *p-values* and HR with 95% CI with the log-rank test and univariate Cox regression.

### Relationship of TBC1D22A with immune checkpoint, stemness index, and drug IC50

The TCGA dataset provided RNAseq data and related clinical data of 376 OSC. SIGLEC15, TIGIT, CD274, HAVCR2, PDCD1, CTLA4, LAG3, and PDCD1LG2 were immune checkpoint-associated genes, and the extraction of the expression values of these 8 genes was made. Heatmaps of gene associations were presented using the R package "pheatmap". The calculation of mRNA-based stemness index (mRNAsi) was made with the OCLR machine learning algorithm constructed by Malta *et al.*^16^, which was next mapped to the [0,1] scope by a linear transformation that subtracted the minimum and divided by the maximum. Immune infiltration was assessed using the "immunedeconv" R package. Using the R package "pRRophetic" and the genomics of drug sensitivity in cancer (GDSC) database to forecast the chemotherapy response for every patient.

### Expression and prognosis of TBC1D22A in pan-carcinoma

We collected RNAseq data and associated clinical data for 33 types of tumors from the TCGA and GTEx databases. Subsequently, a univariate Cox regression analysis was made with the "forestplot" R package. Additionally, we assessed the difference in TBC1D22A expression among different samples through the rank-sum test.

### Statistical analysis

R software, version 4.0.3 (R Core Team, Vienna, Austria) was adopted to make all the above statistical approaches and R packages. Measurements are displayed as mean ± SD. Wilcox test was used to compare two groups of samples, and Kruskal-Wallis test was used to compare three groups of samples. The log-rank test was employed for the comparison of survival disparities. The association between the two variables was described with Sperman analysis. A *p* value of below 0.05 was considered statistically significant.

## Results

### Patient features

RNAseq data (level3) and corresponding clinical information for 376 ovarian tumors were obtained from the Cancer Genome Atlas (TCGA) dataset (https://portal.gdc.cancer.gov/). Based on the expression of TBC1D22A, patients were divided into two groups, and Table 1 displayed their clinical features. The two groups showed no statistically significant differences in race, age, tumor grade and stage. The Sankey diagram of clinical information for the two groups of patients was presented in Supplementary Figure 1.

### Differential expression of TBC1D22A in ovarian cancer and normal tissues

**Figure 1:**
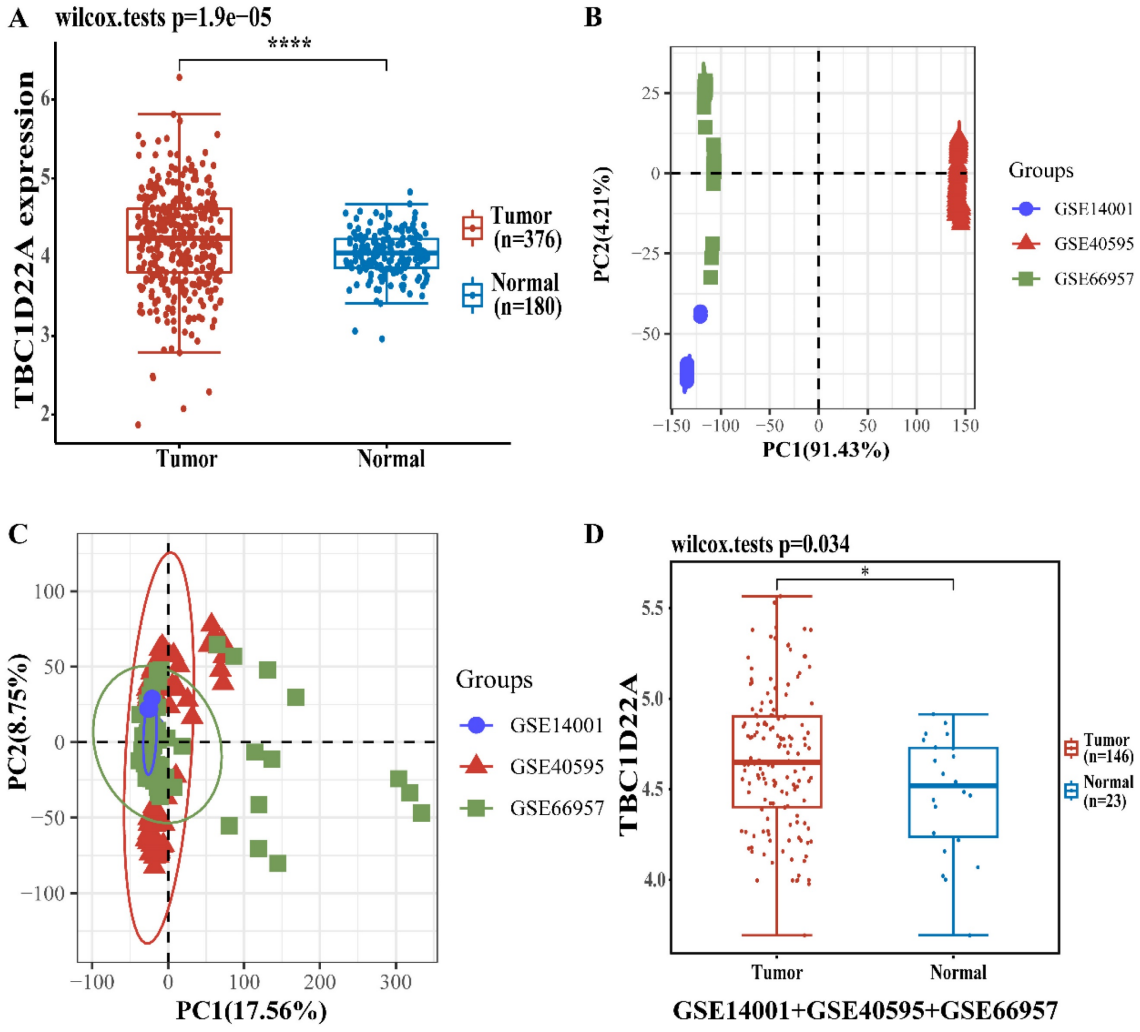
** The different mRNA expressions of TBC1D22A in ovarian cancer and normal tissues. (A)** Expression levels of TBC1D22A in OC and normal tissues. **(B)** Principal component analysis (PCA) results before batch removal for 3 datasets. **(C)** PCA results after batch removal, as shown in the schematic diagram shows the intersection of 3 datasets, which can be used in subsequent analysis. **(D)** The expression of TBC1D22A in datasets GSE14001, GSE40595 and GSE66957 was analyzed integrally. *p < 0.05, ****p < 0.0001.

There were 376 OSC patients in TCGA database chosen as the experimental group, and 180 normal ovaries in GTEx database were selected as the control group. The difference in TBC1D22A expression at the RNA level was analyzed with the R software. Figure 1A displayed the elevated expression of TBC1D22A in ovarian cancer. We then performed an integrated analysis of the 3 datasets (GSE14001, GSE40595 and GSE66957) from the GEO database. And the results indicated that the expression of TBC1D22A was higher in ovarian cancer (Figures 1D), which was consistent with our findings. We then examined the expression of TBC1D22A mRNA in IOSE-80, COC1, ES-2 and SKOV3 cell lines and found higher expression in the COC1, ES-2 and SKOV3 ovarian cancer cell lines than in the normal ovarian cell line IOSE-80 (Figure 3C).

**Figure 2:**
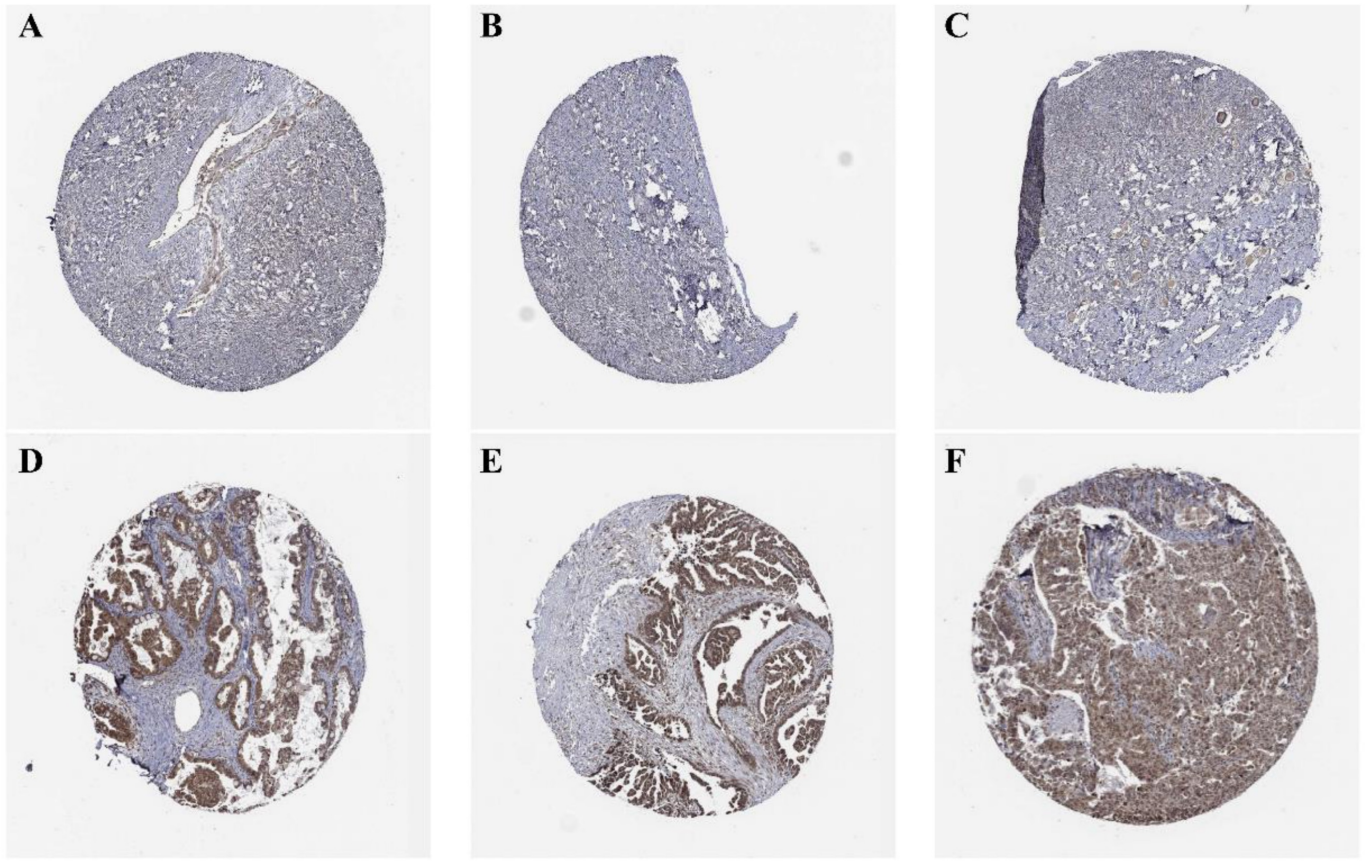
** (A, B, C)** Expression of TBC1D22A in normal ovarian tissue. **(D, E, F)** Expression of TBC1D22A in OSC.

**Figure 3:**
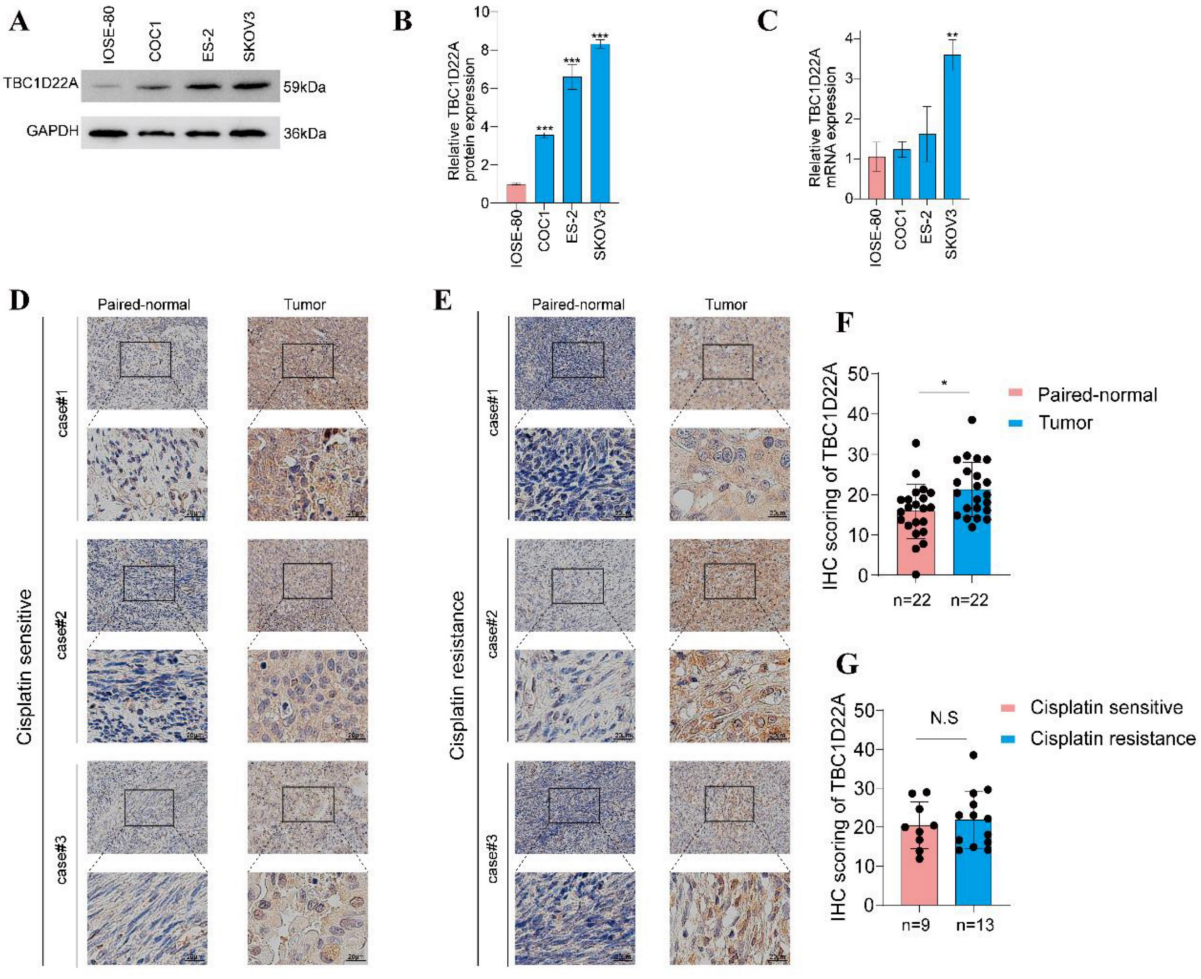
** Differential expression of TBC1D22A. (A)**TBC1D22A protein expression levels in cell lines COC1, ES-2, SKOV3, and IOSE-80. **(B)** Statistical analysis histogram of TBC1D22A gray value. **(C)** TBC1D22A mRNA expression levels in cell lines COC1, ES-2, SKOV3, and IOSE-80. **(D, E)** Immunohistochemical staining of TBC1D22A expression. **(F)** Histogram shows IHC score quantification of TBC1D22A in tumor tissues and adjacent tissues. (G) Histogram shows IHC score quantification of TBC1D22A in tumor tissues of platinum-resistant versus platinum-sensitive patients. *p < 0.05, **p < 0.01, ***p < 0.001, N.S.= not significant.

Immunohistochemical images from the HPA database were analyzed to decide the expression of TBC1D22A at the protein level. The results showed that TBC1D22A was mainly expressed in the cytoplasm and nucleus, which was undetectable in normal ovarian tissues (Figures 2A, B, C) and showed moderate to low intensity staining in tissues of ovarian cancer (Figures 2D, E, F). We subsequently collected 22 pairs of cancer and adjacent tissues for immunohistochemical staining, and the results also showed that TBC1D22A was upregulated in cancer tissues. However, there was no significant difference in the expression between platinum-resistant and platinum-sensitive tumor tissues (Figures 3D, E, F, G). The results of Western blot analysis also suggested that TBC1D22A was overexpressed in ovarian cancer (Figure 3A, B).

### Prognostic value of TBC1D22A among patients with ovarian cancer

**Figure 4:**
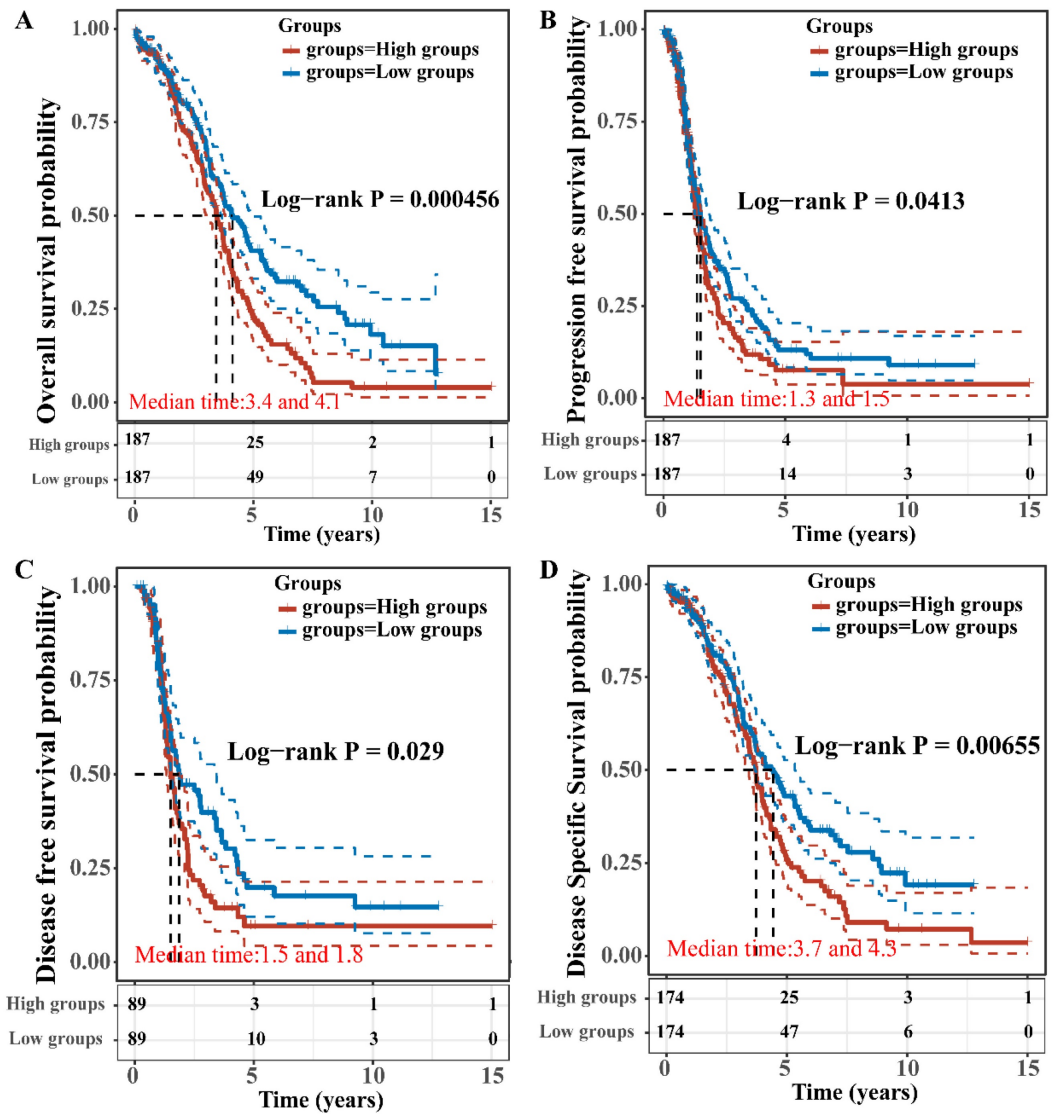
Correlation between TBC1D22A expression and OS **(A)**, PFS (B), DFS **(C)** and DSS **(D)**.

**Figure 5:**
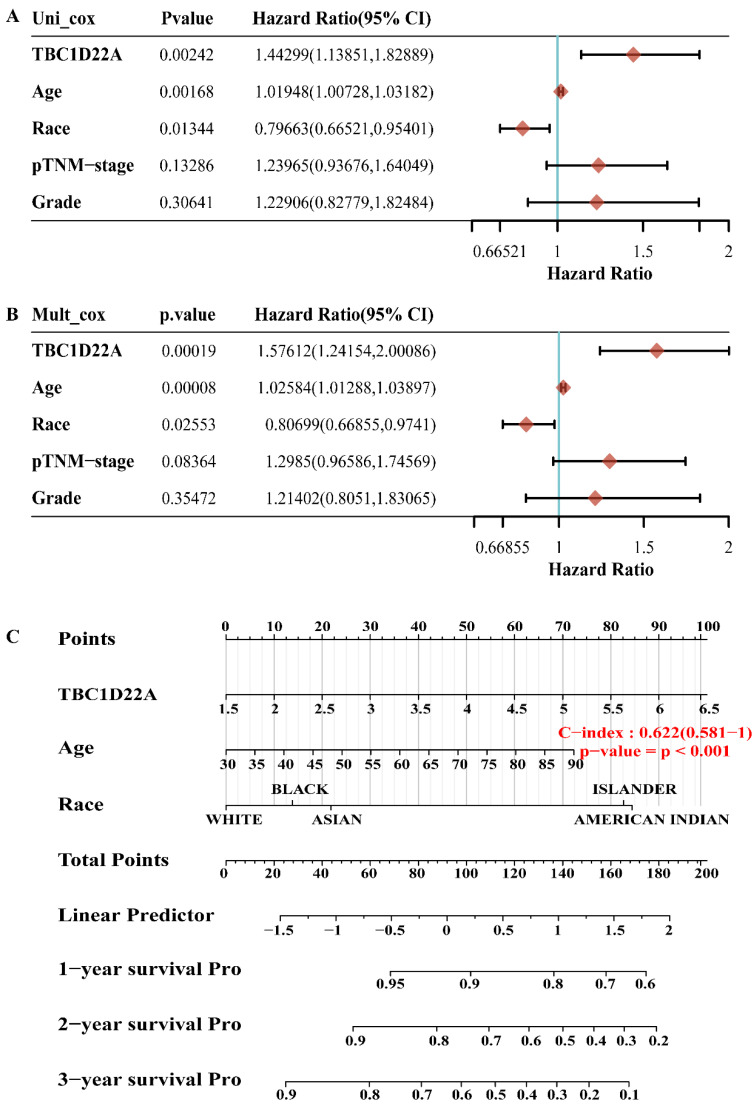
** The *p* value, hazard ratio, and confidence interval are explored by univariate(A) and multivariate(B) Cox regression. (C)** The nomogram predicts 1,2 - and 3-year overall survival of OSC patients.

The OS, PFS, DFS and DSS of patients with high and low expression of TBC1D22A were analyzed to assess the correlation between TBC1D22A expression and the prognosis of ovarian cancer. Significantly, our study revealed that OS (*p* = 0.001), PFS (*p* = 0.05), DSS (*p* = 0.01) and DFS (*p* = 0.05) were notably shorter in patients exhibiting high expression of TBC1D22A compared to those with low expression of TBC1D22A (Figures 4A-D). Another ovarian cancer cohort from the Kaplan-Meier Plotter database again confirmed similar results (Supplementary Figures S2, *p* < 0.001). Subsequently, we conducted univariate and multivariate regression analyzes to delve deeper into the prognostic significance of TBC1D22A in ovarian cancer. In the univariate cox regression analysis, TBC1D22A (HR = 1.44299, *p* < 0.01) and age (HR = 1.01948, *p* < 0.01) appeared as risk factors for the prognosis of patients with ovarian cancer, and race (HR = 0.79663, *p* < 0.05) was a protective factor (Figure 5A). In the next multivariate Cox regression analyses (Figure 5B), TBC1D22A (HR = 1.57612, *p* < 0.001), age (HR = 1.02584, *p* < 0.001), and race (HR = 0.80699, *p* < 0.05) continuously showed predictive value for patients with OSC. Based on the variables affecting the prognosis of patients, Figure 5C predicted the OS of patients at 1, 2, and 3 years in the form of a nomogram. By analyzing the pan-cancer data (Supplementary Figures S3, S4), it was found that TBC1D22A also has a prognostic value in esophageal carcinoma (ESCA) and liver hepatocellular carcinoma (LIHC), which needs further research in related fields.

### Correlation of TBC1D22A with immune checkpoint, stemness index, and drug IC50

**Figure 6:**
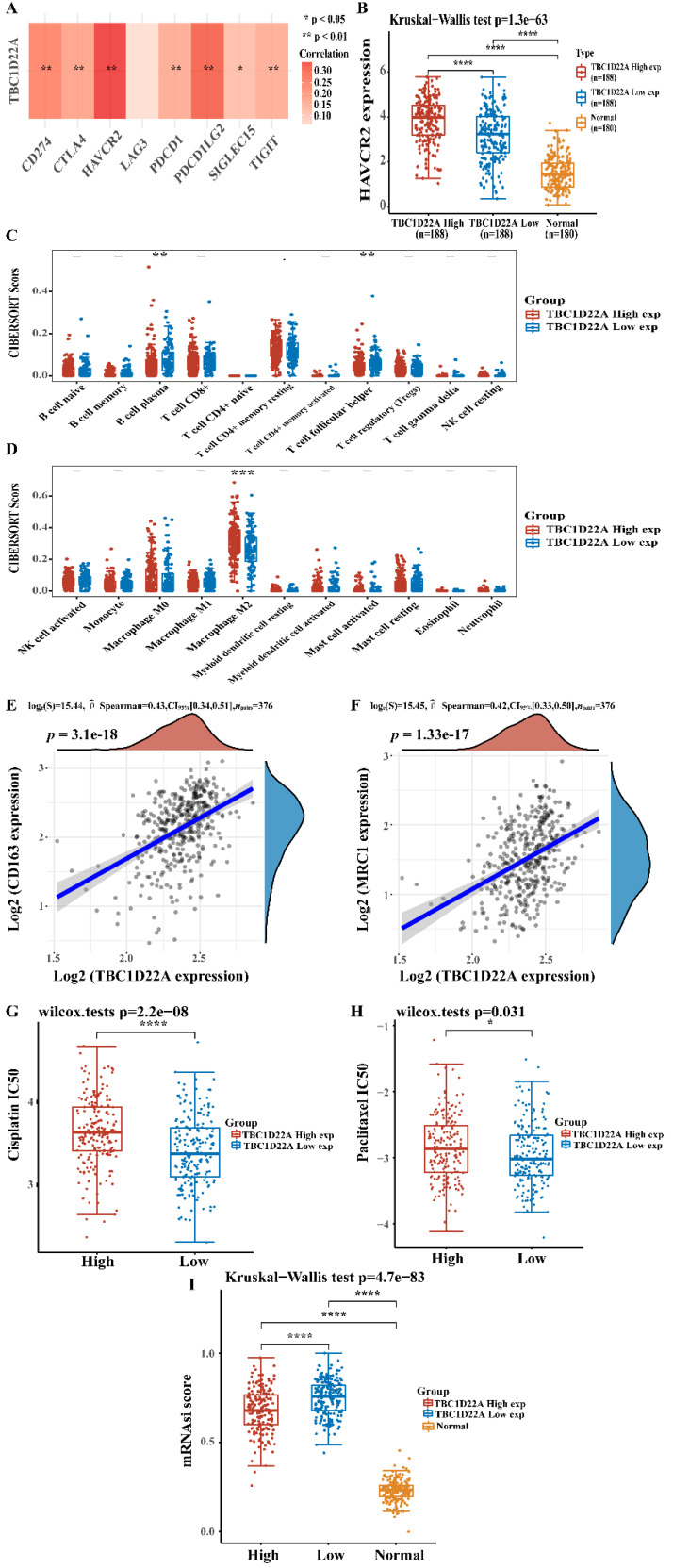
** (A) TBC1D22A and immune checkpoint expression heat map. (B)** HAVCR2 was expressed at higher levels in patients with OSC who had high TBC1D22A expression. **(C, D)** Association between TBC1D22A expression and immune cell infiltration. **(E, F)** Association between TBC1D22A expression and M2 macrophage markers CD163 and MRC1. **(G, H)** Correlation between TBC1D22A expression and IC50 scores of cisplatin and paclitaxel. **(I)** Correlation between TBC1D22A expression and stemness index.

To explore the mechanism of the poor prognosis of OSC caused by the high expression of TBC1D22A, the relationship between TBC1D22A and immune checkpoints and immune infiltration was analyzed. Figure 6A showed a strong positive correlation between TBC1D22A and the immune checkpoints HAVCR2, PDCD1LG2 and CD274 (*p*<0.01). HAVCR2 expression was higher in the TBC1D22A high expression group compared to the low expression group (Figure 6B). According to the immune infiltration analysis, the content of M2 macrophages in patients with high TBC1D22A expression was significantly increased (*p* < 0.001) (Figure 6D). Spearman correlation analysis also confirmed that TBC1D22A was significantly and positively associated with M2 macrophage markers CD163(*p* < 0.001) and MRC1(*p* < 0.001) (Figures 6E, F). These findings suggested that TBC1D22A may contribute to tumor immune escape by affecting macrophage polarization. Moreover, patients with high expression of TBC1D22A had a higher IC50 of cisplatin (*p* < 0.001) and paclitaxel (*p* < 0.05) (Figures 6G, H) and were more likely to develop drug resistance. Figure 6I showed that ovarian cancer patients in the high expression TBC1D22A group showed a lower stemness index than those in the low expression TBC1D22A group (*p* < 0.001). For a higher expression of TBC1D22A, tumor cells were likely to be well-differentiated.

## Discussion

TBC family proteins are correlated with the occurrence and development of various tumors. Rab is an evolutionarily conserved small GTPases in eukaryotic cells, which mainly participates in a series of intracellular transport processes such as membrane vesicles formation, movement of membrane vesicles to target organelles, and binding of membrane to specific target membrane^17^. The role of TBC/Rab-GAPs in malignant tumors may be directly related to its regulation of Rabs, which can promote the transport and recycling of receptor proteins from cell to cell and cell to matrix, thereby causing cell invasion. Furthermore, many components of oncogene signaling, such as EGFR, RAS, and RAC, are localized to endocytic vesicles or need to be properly transported to the cell membrane or adhesion sites^18^,^19^. TBC1D3 was a GAP for Rab5, which was up-regulated in metastatic prostate cancer. Overexpression of TBC1D3 can drive the growth of NIH3T3 cells *in vitro* or *in vivo* and promote the loss of contact inhibition phenomenon. The tumor-promoting effect of TBC1D3 was abolished when the GAP domain mutation of TBC1D3 was inactivated^20^. Bioinformatics analysis showed that TBC1D16 was a melanoma driver gene and could promote the development of melanoma cells^21^. The high expression of TBC1D7, a GAP for Rab17, was found in lung cancer tissues, which can significantly drive the development of lung cancer cells and was correlated with poor prognosis of patients^22^. For all we know, this research first reports the expression and prognostic value of TBC1D22A in ovarian cancer.

Recently, in addition to platinum-based combined chemotherapy, bevacizumab^23^ and poly (ADP-ribose) polymerase inhibitors^24^ have provided new options for treating ovarian cancer. However, these new treatments do not seem to have significantly improved the prognosis for advanced ovarian cancer.

Therefore, more prognostic markers and therapeutic targets need to be sought to improve the outcomes of patients. The current research first demonstrated the high expression of TBC1D22A in ovarian cancer. Patients with elevated expression of TBC1D22A show a poor outcome. Subsequently, through a comprehensive exploration of the patient's clinical characteristics, it was proved that TBC1D22A was an independent prognostic factor for ovarian cancer. The possible mechanism by which TBC1D22A affected patient outcomes was further investigated. Recently, immunotherapy represented by checkpoint blockade has gradually emerged in the treatment of tumors and has made significant progress in various solid tumors^25^. However, clinical studies have shown that ovarian cancer is not sensitive to checkpoint blockade, and only 6%-22% of patients respond to programmed death-1 (PD-1) and PD-L1 antibody treatment^26^,^27^. Research on immuno-suppressive genes and their mechanisms is essential for selecting patients suitable for checkpoint blockade and for using combination therapy to improve the response rate to checkpoint blockade immuno-therapy. M2 macrophages can promote tumor proliferation and metastasis^28^,^29^. Our findings displayed that TBC1D22A was co-expressed with multiple immune checkpoints, including HAVCR2, PDCD1LG2, CTLA4, PDCD1(PD1), TIGIT, SIGLEC15, and CD274(PD-L1). Immune cell infiltration analysis showed that TBC1D22A could promote the enrichment of M2 macrophages in ovarian cancer tissues to exert an immunosuppressive effect, and ultimately promoted the progression of ovarian cancer. Therefore, combined use of TBC1D22A inhibitors may improve the effect of immune checkpoint blockade on ovarian cancer, and TBC1D22A may also act as a molecular marker for screening whether immune checkpoint blockade can be used. Platinum-based chemotherapy agents and paclitaxel are first-line chemotherapy agents for ovarian cancer. In this study, it was more likely that patients with high expression of TBC1D22A develop drug resistance, which may be a reason for the poor prognosis of patients.

In fact, this study has certain limitations and more *in vivo* and *in vitro* experiments are necessary for revealing the carcinogenic mechanism of TBC1D22A in ovarian cancer, which we are planning to do in the future. In addition, it would be more perfect if a large number of single or multi-center clinical follow-up data were available to further verify the prognostic value of TBC1D22A.

## Conclusion

In conclusion, the expression of TBC1D22A in ovarian cancer and its relationship with prognosis, immune infiltration, and IC50 of first-line chemotherapy drugs were analyzed in an integrated way. The findings indicated that elevated TBC1D22A expression was correlated with a poor outcome in ovarian cancer and served as a significant independent prognostic indicator. We need to further study the carcinogenic mechanism and clinical application of TBC1D22A in ovarian cancer.

## Supplementary Material

Supplementary figures and tables.

## Figures and Tables

**Table 1:** Clinical characteristics of patients with high and low expression of TBC1D22A.

Characteristics		High TBC1D22A express	Low TBC1D22A express	*p*-value
Age	Mean (SD)	58.6 (10.9)	60.6 (11.7)	
	Median [MIN, MAX]	58 [30, 83]	60 [34, 87]	0.073
Race	AMERICAN INDIAN	2		
	ASIAN	5	6	
	BLACK	14	11	
	ISLANDER	1		
	WHITE	160	166	0.772
pTNM stage	IC	1		
	IIA	2	1	
	IIB	1	2	
	IIC	9	6	
	IIIA	2	5	
	IIIB	7	7	
	IIIC	136	136	
	IV	28	30	0.856
Grade	G1	1		
	G2	23	19	
	G3	158	164	
	GX	5	3	
	G4		1	0.763
New tumor event type	Progression	7	11	
	Recurrence	106	93	0.356
Radiation therapy	Non-radiation	2	4	
History of neoadjuvant	Neoadjuvant	1		
	No neoadjuvant	187	188	

## Abbreviations

TBC1D22A: TBC1 domain family member 22A
GAP: GTPase-activating protein
OSC: ovarian serous cystadenocarcinoma
PFS: progression free survival
DSS: disease specific survival
OS: overall survival
TCGA: The Cancer Genome Atlas database
GTEx: genotype-tissue expression database
RT-qPCR: reverse transcription-quantitative polymerase chain reaction
HR: hazard ratio
CI: confidence interval
