# Supplementary material for: High Expression of TBC1 Domain Family Member 22A is Related to Poor Prognosis in Ovarian Serous Cystadenocarcinoma
Source: Int J Med Sci. 2024 Oct 7;21(13):2603–12. doi: 10.7150/ijms.99744 (PMC11492882; doi:10.7150/ijms.99744)
Supplement: Supplementary file 1 — Supplementary figures and tables. [file ijmsv21p2603s1.pdf]

Supplementary Material

Supplementary Figures

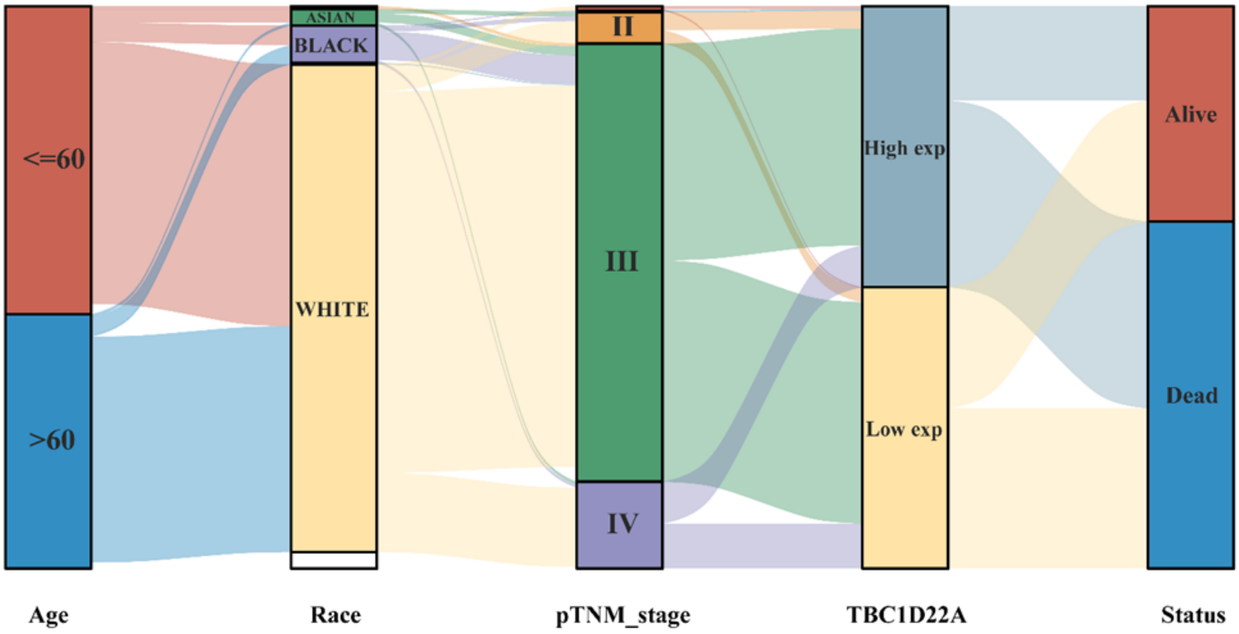

Supplementary Figure 1. The Sankey diagram of TBC1D22A and clinical characteristics. Each row represents a feature variable, different color represents different typing or stage, lines represent the distribution of the same sample in different feature variables.

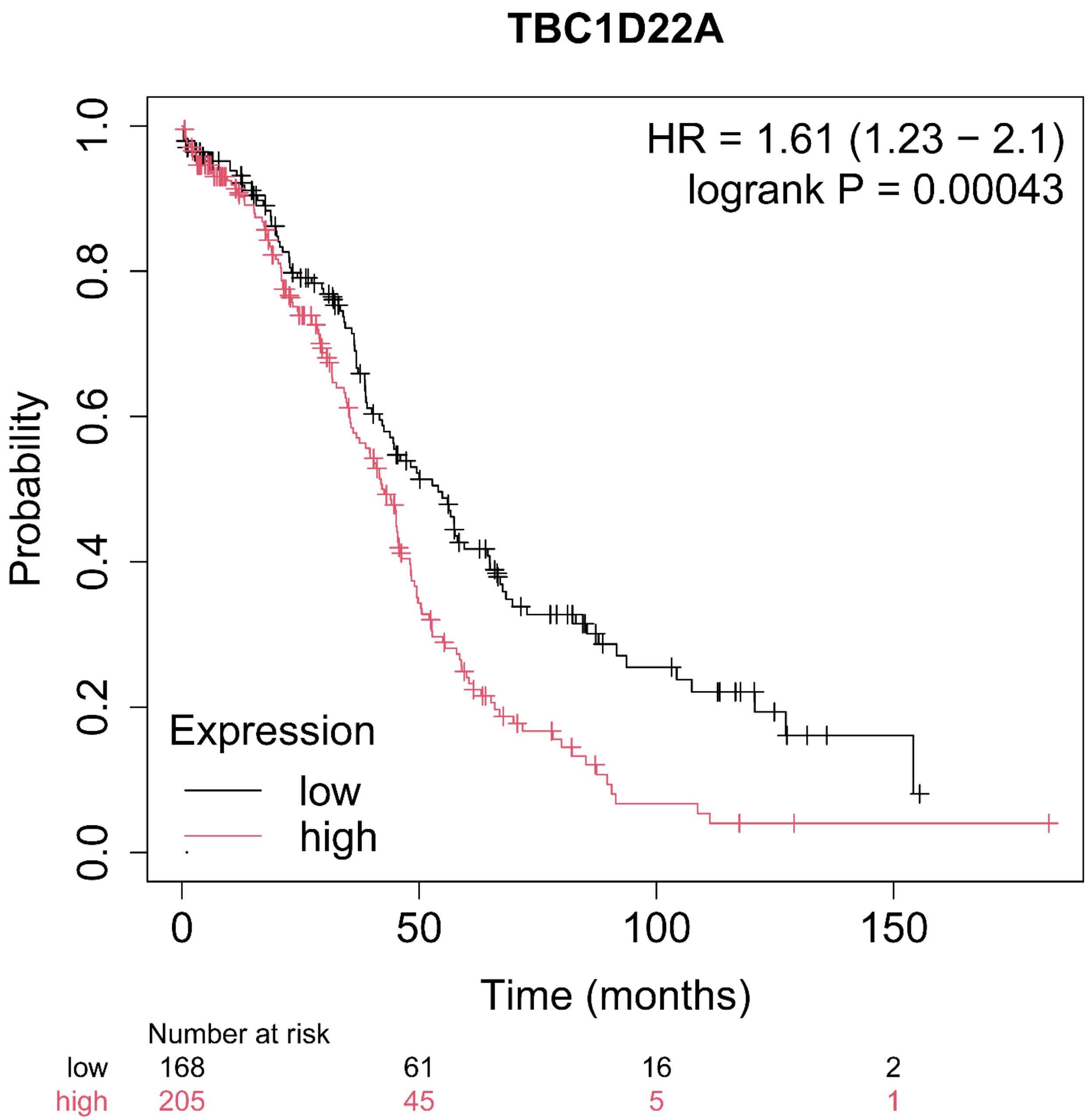

Supplementary Figure 2. Kaplan-Meier Plotter database verified the correlation between TBC1D22A and OS.

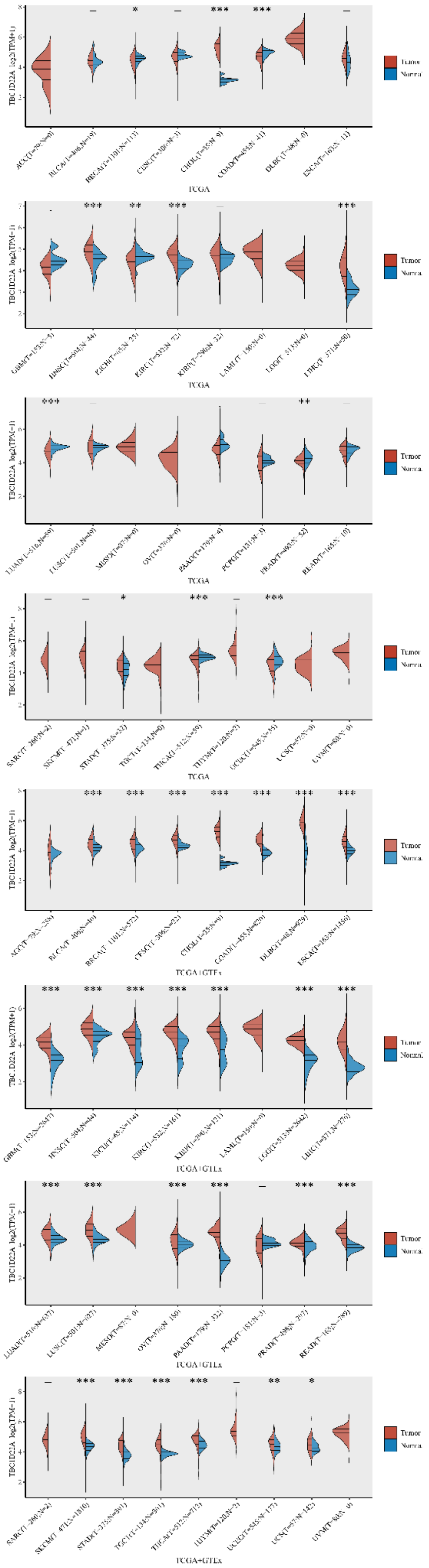
TBC1D22A log2(TPM+1)
Tumor
Normal
TCGA
TCGA+GTEx

Supplementary Figure 3. Differential expression of TBC1D22A in pan-carcinoma.

Cancer	Pvalue	Hazard Ratio(95% CI)
ACC	0.0297	2.36588(1.08854,5.14207)
BLCA	0.1687	1.23058(0.91576,1.65364)
BRCA	0.5450	1.10317(0.80271,1.51609)
CESC	0.2072	0.74177(0.46633,1.17989)
CHOL	0.9328	1.04091(0.41,2.64269)
COAD	0.7757	1.05809(0.7175,1.56036)
DLBC	0.7149	1.31085(0.30669,5.60285)
ESCA	0.0353	0.57832(0.34731,0.96297)
GBM	0.7957	1.04851(0.73257,1.50071)
HNSC	0.6078	0.93254(0.71419,1.21764)
KICH	0.2684	2.19198(0.54611,8.79819)
KIRC	0.2811	0.84878(0.62996,1.14362)
KIRP	0.0510	0.54141(0.29231,1.00279)
LAML	0.8584	0.96215(0.62963,1.47028)
LGG	0.3112	1.19991(0.84327,1.70739)
LIHC	0.0102	1.57841(1.11403,2.23637)
LUAD	0.7426	1.04986(0.7853,1.40354)
LUSC	0.6530	1.06413(0.81155,1.39531)
MESO	0.8633	0.95914(0.59652,1.5422)
OV	0.0005	1.60027(1.23036,2.08141)
PAAD	0.5032	1.14944(0.76451,1.7282)
PCPG	0.0908	0.16028(0.0192,1.33775)
PRAD	0.5936	0.70663(0.19738,2.52969)
READ	0.1179	0.51119(0.22042,1.18552)
SARC	0.4830	1.15214(0.7756,1.71149)
SKCM	0.7837	1.03826(0.79414,1.35741)
STAD	0.9521	0.98999(0.71293,1.37474)
TGCT	0.3306	0.32514(0.03382,3.12616)
THCA	0.2605	0.55905(0.20302,1.53945)
THYM	0.7343	1.25774(0.33449,4.7294)
UCEC	0.3560	0.82215(0.54246,1.24604)
UCS	0.9713	1.01234(0.51914,1.97408)
UVM	0.8051	1.10982(0.48503,2.53947)

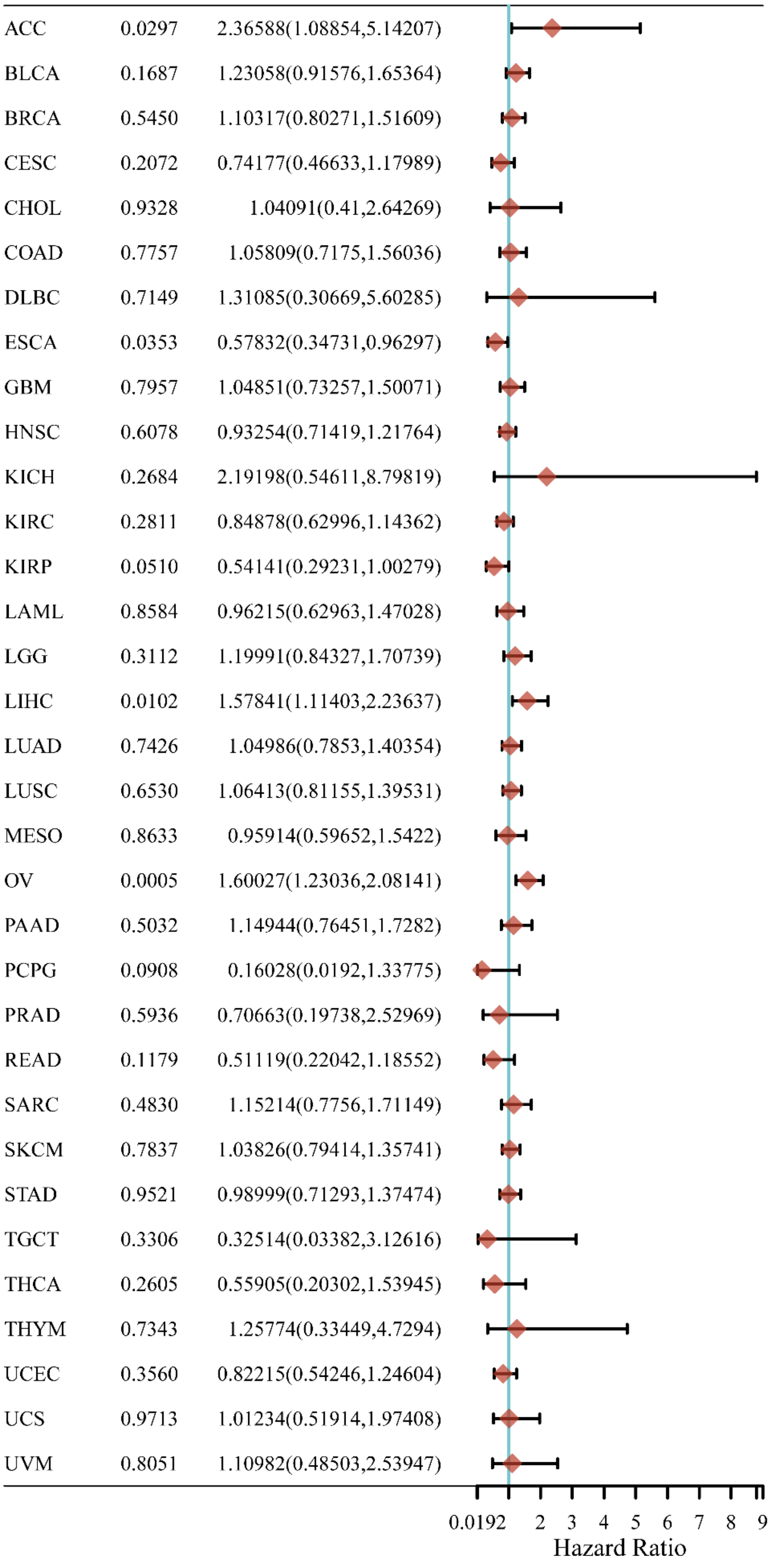

Supplementary Figure 4. Univariate cox regression analysis of TBC1D22A in pan-carcinoma.
